# Direct experimental observation of weakly-bound character of the attached electron in europium anion

**DOI:** 10.1038/srep12414

**Published:** 2015-07-22

**Authors:** Shi-Bo Cheng, A. W. Castleman

**Affiliations:** 1 Department of Chemistry, The Pennsylvania State University, University Park, Pennsylvania 16802, United States; 2 Department of Physics, The Pennsylvania State University, University Park, Pennsylvania 16802, United States

## Abstract

Direct experimental determination of precise electron affinities (EAs) of lanthanides is a longstanding challenge to experimentalists. Considerable debate exists in previous experiment and theory, hindering the complete understanding about the properties of the atomic anions. Herein, we report the first precise photoelectron imaging spectroscopy of europium (Eu), with the aim of eliminating prior contradictions. The measured EA (0.116 ± 0.013 eV) of Eu is in excellent agreement with recently reported theoretical predictions, providing direct spectroscopic evidence that the additional electron is weakly attached. Additionally, a new experimental strategy is proposed that can significantly increase the yield of the lanthanide anions, opening up the best opportunity to complete the periodic table of the atomic anions. The present findings not only serve to resolve previous discrepancy but also will help in improving the depth and accuracy of our understanding about the fundamental properties of the atomic anions.

Owing to the existence of abundant unpaired *f* electrons, lanthanide elements form a very important group in the periodic table, which are highly valuable to many modern technologies, including clean energy, consumer electronics, and advanced transportation, etc. Unlike main group elements, however, the knowledge of the fundamental physicochemical properties of the lanthanides is extremely limited, which hinders the complete understanding about the properties of atoms. Specifically, one of the greatest concerns that has puzzled the experimentalists and theoreticians for several decades is the electron affinities (EAs), which can be viewed as one of the most important properties in ionic chemistry^1^,^2^,^3^, of the lanthanide atoms. Note that, as the simplest systems, research on the EAs of atoms can be traced to 1950s and 1960s^4^,^5^,^6^. Pioneered by Branscomb *et al*., the photodetachment of the atomic H^−^ and D^−^ have been performed in a modulated crossed-beam experiment^4^. Subsequently, Lineberger and co-workers made a significant contribution to this scientific field. Advanced dye lasers have been employed by them to measure the EAs of elements via high-resolution threshold photodetachment spectroscopy^7^,^8^, enabling them to obtain the total photodetachment cross sections of a series of atomic anions. Although the negative ion properties of many elements have been reported^9^,^10^,^11^,^12^, the information about experimental EAs of the lanthanide atoms is still limited, or even conflictive with respect to the theoretical predictions.

It is well-accepted that the experiments and theoretical calculations on lanthanides are particularly challenging. Theoretically, the large number of electrons and presence of open shells (*d* and/or *f*) in lanthanides result in extremely complicated calculations on the electronic structures of these heavy elements^12^,^13^. Experimentally, it is quite challenging to produce sufficient anions that can be used in the photodetachment experiments. This situation is especially true for most of the lanthanide anions except La^−^ and Ce^−^ since the yields of the latter were found three orders of magnitude greater than those of other lanthanide anions^14^. In early 2000s, a series of measurements of EAs of lanthanides were attempted by Davis and Thompson, including Eu^15^, Tm^16^, and Pr^17^. EAs of ~1 eV were reported for these lanthanides, implying a relatively strong interaction between the extra electron and the neutral. These findings were considered as a breakthrough in atomic negative ions field. Subsequent high-level theoretical calculations, however, raised questions about these measurements^18^,^19^,^20^. Theoretical EAs of most lanthanides are only dozens or hundreds of meV, much smaller than previous experimental results. In some cases, the experimental EAs are one order of magnitude larger than theory, *e.g*., Eu. The EA of Eu was measured to be 1.053 ± 0.025 eV (strongly-bound)^15^, while the theoretical values are about 0.117 and 0.116 eV (weakly-bound), respectively^18^,^20^. Note that only a rough lower limit of the EA of Eu (≥0.05 eV) was estimated by Nadeau *et al*. due to the limitations of the experimental technique^14^. Such a significant discrepancy between experiment and theory clearly shows the challenge in obtaining accurate EAs of lanthanides. It is necessary to mention that some recent studies measured the EA of another lanthanide, Ce, whose yield of the anion is much higher than that of Eu^−14^. Although Davis and Thompson suggested a 0.955 ± 0.026 eV EA for Ce based on their LPES experiment^21^, a subsequent reinterpretation of the LPES data claimed an EA of 0.660 eV^22^, which is consistent with later experimental results along with the theoretical predictions^19^,^23^,^24^,^25^,^26^. Thus, no significant discrepancy exists in Ce, which is completely different from the present case, Eu. As for Eu, therefore, a central and important question is: can we increase the yield of Eu^−^ to a detectable level and then understand the true interaction between the additional electron and the neutral in Eu^−^ ion?

We explored this question by utilizing the photoelectron spectroscopy, which has been proven to be a powerful approach to directly probe the electronic properties of atoms and clusters^27^,^28^,^29^,^30^,^31^,^32^,^33^,^34^,^35^,^36^,^37^,^38^,^39^,^40^. Herein, we present direct experimental observations on the features of the electron-atom interaction in Eu^−^. The EA was measured to be 0.116 ± 0.013 eV, representing a weakly-bound character between the extra electron and Eu, which is in outstanding agreement with recently reported high-level theoretical calculations with the values of 0.117 and 0.116 eV, respectively^18^,^20^. The present finding reveals the first precise experimental EA of Eu, clearly eliminating the longstanding discrepancy in previous experiment and theory. Also, the new experimental strategy proposed herein has been found successful in producing detectable lanthanide anions, providing the best opportunity in completing the periodic table of the negative ions.

## Results

### Mass spectrum of the europium (Eu) anion

**Figure 1:**
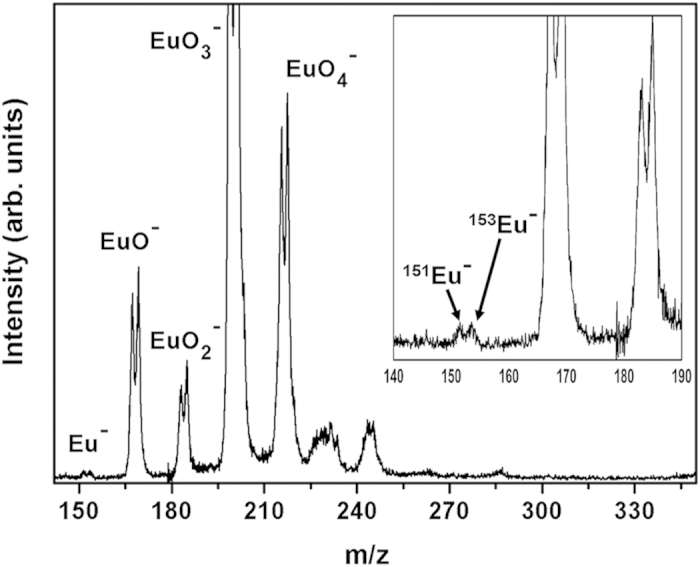
Time-of-flight mass spectrum of Eu^−^ and monoeuropium oxide clusters. The inset shows the enlarged spectrum in the range of 140 to 190 m/z.

The greatest challenge hindering the attainment of correct EAs of most of the lanthanides is the difficulty of producing sufficient anions that can be used in the photodetachment experiments utilizing conventional experimental method. In photodetachment experiments, helium (He) or argon (Ar) is widely used as an effective expansion or cooling gas to produce pure atomic or cluster anions^41^. For example, Ce^−^ can just be generated by using such experimental method^25^. However, in the case of Eu^−^, employing these conventional carrier gases did not produce any detectable atomic anions, probably because the yield of Eu^−^ is much lower than that of Ce^−^14. In one of our recent studies, it has been established that the addition of N_2_O into helium is beneficial to produce smaller oxide clusters, *e.g*., MgO^−^40. Thus, a possible strategy for synthesizing Eu^−^ is proposed as follows: utilizing N_2_O+ He as a reactant gas to increase the yield of EuO^−^ followed by increasing the output of the ablation laser to provide sufficient energy to open the reaction channel dissociating EuO^−^ into Eu^−^ and O. Figure 1 displays the mass spectrum of the EuO_x_^−^ (x = 0–4) clusters using the abovementioned method. It was found that the intensity of the Eu^−^ signal is very sensitive to the power of the ablation laser, and the Eu anion can only be observed at high laser power. The inset in Fig. 1 is an enlarged spectrum in the range of 140 to 190 m/z to clearly show the peak distribution of Eu^−^, in which two isotopes at 151 and 153 amu are evidenced. The assignment of the Eu^−^ peak is validated from both the mass-to-charge ratio and the isotopic distribution. It is worth noting that there may exist another dissociation channel, *e.g*. EuO^−^ → Eu + O^−^, which is probably more favorable than the suggested channel forming Eu^−^ since atomic O has a higher EA than that of Eu. This could be deduced from the relative low intensity of the Eu^−^ peak observed here, as shown in Fig. 1. However, the encouraging experimental fact is that, as will be shown in the following section, we were able to acquire the photoelectron image of the Eu^−^ by photodetaching the experimentally produced anions. This indicates that the experimental strategy used here successfully increased the yield of the Eu^−^ ion to a detectable level, which could be viewed as a significant advance in producing the gas-phase lanthanide anions. These findings open up great opportunity for us to correctly understand the fundamental properties of these heavy *f*-block atoms.

### Photoelectron imaging spectroscopy and EA of Eu

**Figure 2:**
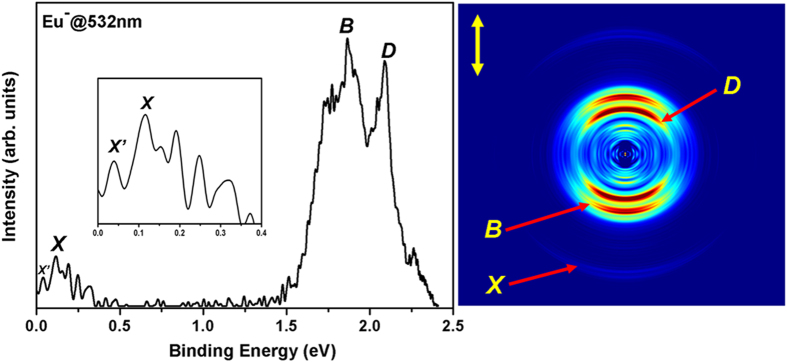
Photoelectron image and corresponding photoelectron spectrum of Eu^−^. The spectrum was obtained at 532 nm photon energy. The inset shows the enlarged spectrum in the range of 0 to 0.4 eV.

Figure 2 depicts the photoelectron image and corresponding binding energy spectrum of Eu^−^ obtained at 532 nm photon energy. The double yellow arrow represents the direction of the laser polarization. As shown in Fig. 2, three prominent rings can be identified, labeled X, B and D. Careful inspection of the spectrum reveals many other resolved peaks at the binding energy range of 1.63–2.30 eV, which will be discussed below. The weak ring X appears at the very edge of the camera, implying an extremely low binding energy for this transition. Among the observed peaks in the photoelectron spectrum, the ones lying at low binding energy region (up to 0.4 eV) are more interesting since they contain the EA defined transition. To clearly show the peak distributions of this region, an enlarged spectrum is included as an inset in Fig. 2. As shown in the inset of Fig. 2, peak X is the most intense transition in the low binding energy region, and the measured binding energy is 0.116 ± 0.013 eV. In most of the photodetachment process, it is generally accepted that, among adjacent transitions, the peak with the greatest intensity results from transition between lowest-lying levels^42^. It is, therefore, reasonable to temporarily assign X as the EA defined peak coming from the transition between the ground state of Eu^−^ to the corresponding neutral ground state, and the EA of Eu is 0.116 ± 0.013 eV.

In order to validate the above identification, we have compared the energy spacings of the observed peaks (Fig. 2) with well-known Eu neutral spectrum^43^, which can provide the most straightforward and strongest support for our assignment of the EA defined peak. This is also the reason that we utilized 532 nm (2.33 eV) laser wavelength to detach Eu^−^, which is energetically accessible to generate neutral Eu in excited states, while the 1064 nm (1.17 eV) photon energy is not sufficient to produce excited Eu neutral. Recently, Beck *et al*. theoretically suggested that the photodetachment channels from anionic ground state to ^10^P_7/2_ and ^8^P_5/2_ neutral thresholds of Eu will produce stronger peaks located at 1.862 and 2.088 eV, respectively, in the photoelectron spectrum^18^. This prediction is nicely reproduced in our spectrum (Fig. 2) since the two strongest transitions B and D appear at 1.864 and 2.088 eV, respectively. Therefore, considering the energies of these two transitions and the known term energies from previous atomic absorption spectroscopy^43^, the EA of Eu can be calculated to be about 0.119 eV, which is closer to the suggested EA defined peak (X) than any other adjacent peaks in the low binding energy region. This provides the first experimental evidence that our assigned EA defined peak (X) is reliable.

**Figure 3:**
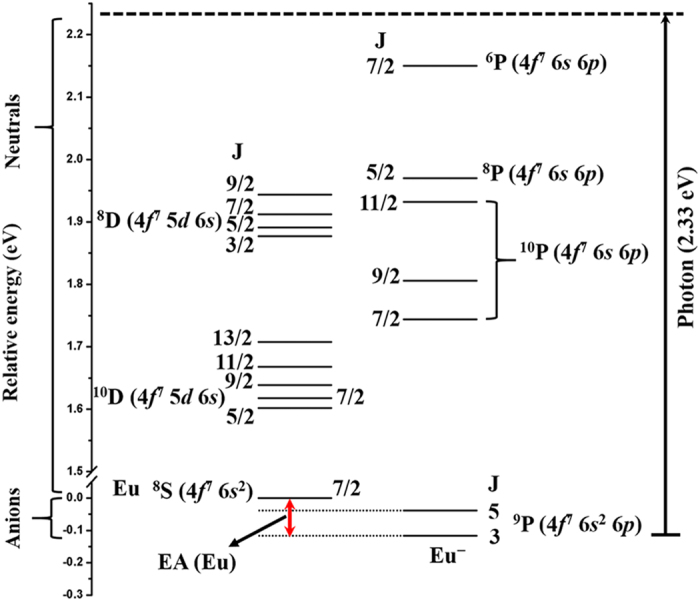
Schematic of energy levels in neutral and anionic Eu observed in the experiment. The energy levels of the neutral Eu are obtained from Ref. 43, while those of the anionic Eu are acquired from the present experiment. The EA of Eu is defined as the energy difference between the lowest energy levels of the neutral and the anion. Leading electronic configurations and LS terms are included.

**Figure 4:**
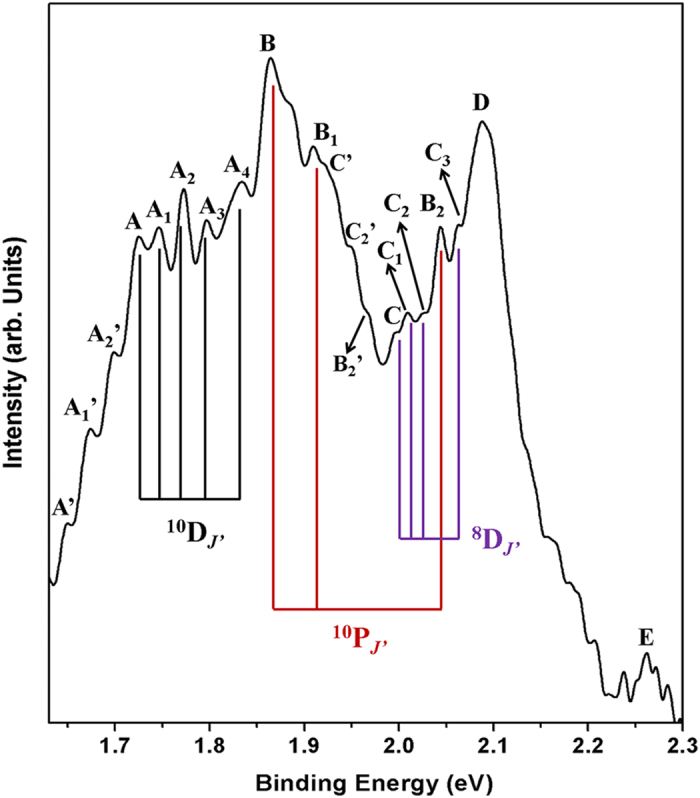
Enlarged photoelectron spectrum of Eu^−^ between 1.63 and 2.30 eV. Transitions from anionic ground state to different excited states of neutral Eu atom are labeled using different letter series (A_*i*_, B_*i*_, …), and are indicated by different color lines. Transitions from one excited anionic state are marked with apostrophe.

Figure 3 shows the energy levels of neutral Eu^43^ with those of the Eu anion sketched in. As shown in Fig. 3, photodetachment with 532 nm photon energy will raise the energy of the ground-state Eu anion by 2.33 eV to an energy level from which it will be able to eject an electron. Taking the suggested EA value (0.116 ± 0.013 eV) of Eu into account, the absorbed photon energy is capable of producing neutral atom either in ground state (^8^S_7/2_) or in one of several excited states (^10^D_*J’*_, ^10^P_*J’*_, ^8^D_*J’*_, ^8^P_*J’*_, or ^6^P_*J’*_). Therefore, the PES can be expected to consist of six groups of peaks. Moreover, the *j*-level fine structure resulting from the spin-orbit splitting of these levels should produce structures in each of these peaks. To observe the fine structures clearly, the higher binding energy region (1.63–2.30 eV) of the spectrum (Fig. 2) has been enlarged, and is shown as Fig. 4. Note that all peaks in Fig. 4 represent the transitions between the anionic Eu level and the excited states of neutral Eu. As shown in Fig. 4, five groups of peaks are observed, labeled as A_*i*_, B_*i*_, C_*i*_, D, and E, respectively, corresponding to the transitions to different excited states of neutral Eu. These fine structures allow us to further verify the assignment of the EA defined peak suggested here by comparing them with the known neutral excited-state term energies^43^. In Table 1, the binding energies of different peaks are listed along with the energy levels of neutral Eu extracted from the present measurements. The known excited states of neutral Eu are also summarized for comparison^43^. As shown in Table 1, good agreement between the present measurements and the well-established electronic structures of neutral Eu^43^ is evidenced with the maximum deviation of only 0.018 eV, giving us further confidence that our assignment of the EA defined peak (X) is correct. Note that the electron configuration of the ground-state Eu^−^ is 4*f*^  7^ 6*s*^2^ 6p (vide infra). And, according to the electronic structures of Eu^43^, the electron configurations for the final neutral excited states corresponding to the peaks B_*i*_, D and E are 4*f*^  7^ 6*s* 6*p*, while those of the peaks A_*i*_ and C_*i*_ are 4*f*^  7^ 5*d* 6*s*. Thus, the peaks B_*i*_, D and E could occur from direct photodetachment of a 6*s* electron. In the case of peaks A_*i*_ and C_*i*_, they may be formed via multi-step processes. Here, we provide one possible explanation about the formation of peaks A_*i*_ and C_*i*_, which is as follows: the absorption of the photon energy (2.33 eV) may promote the ground-state Eu^−^ ion to an excited anionic state probably with a 4*f*^  7^ 5*d* 6*s*^2^ electron configuration followed by a 6*s* orbital detachment to form the final neutral thresholds since the photoelectron angular distributions (PADs) of these peaks (see Fig. 2) are preferably oriented parallel to the laser polarization, which imply that the photoelectron detachment occurs from atomic orbital of a mainly *s*-type character. Lastly, Beck *et al*. suggested that the cross sections of the 4*f*^  7^ 6*s*^2^ 6*p* → 4*f*^  7^ 6*s* 6*p* channels should be much larger than those in the 4*f*^  7^ 6*s*^2^ 6*p* (electron configuration of ground-state Eu^−^) → 4*f*^  7^ 6*s*^2^ (electron configuration of ground-state Eu) photodetachment channels^18^, which is also evidenced in the present experiments since the intensities of peaks B and D (*s*-electron detachment transitions) are much stronger than that of the *p*-electron detachment band (X) (Fig. 2). Therefore, based on all these findings, it is reasonable to conclude that the peak X in Fig. 2 represents the transition from the ground state of Eu^−^ to the corresponding neutral ground state, and the EA of Eu is determined to be 0.116 ± 0.013 eV. Additionally, it is necessary to mention that the electron configuration of the ground state of Eu^−^ should be 4*f*^  7^ 6*s*^2^ 6*p* (^9^P_3_) since the measured EA of Eu is in excellent agreement with the theoretical value calculated by Beck *et al*. with the basic assumption that it is the *p*-electron attachment leading to the formation of ground-state Eu^−^ from neutral Eu (4*f*^  7^ 6*s*^2^) atom^18^.

It is apparent that the newly established EA value (0.116 ± 0.013 eV) of Eu here differs considerably from Davis and Thompson’s result (1.053 ± 0.025 eV)^15^, but is in outstanding agreement with recently reported theoretical predictions^18^,^20^. We believe our EA (0.116 ± 0.013 eV) obtained here is more reliable since, based on the above discussions, the present measurement is not only consistent with the high-level calculations^18^,^20^ but also in excellent agreement with the well-established neutral electronic structures of Eu^43^. It was suggested that the significant overestimation in previous measurement^15^ may originate from following reasons: (a) the transition is likely from the anionic ground state to the excited state of neutral, or (b) from long-lived metastable states of anion to the excited state of Eu^18^. The first possibility can be easily ruled out since no peaks were found around 1 eV in our PES. Thus, one possible explanation for the overestimation in previous measurement is that the produced anions were not in their ground state, and the observed peaks may originate from transitions between the anionic metastable states and the excited states of Eu. To verify this suggestion, more accurate theoretical methods are urgently desired to quantitatively locate the relevant excited states of Eu^−^. Additionally, another possibility is that the photodetached species were other species rather than the Eu^−^ ion, *e.g*. EuH^−^. To testify this assumption, photodetachment experiments on EuH^−^ need to be done and compared with previous spectrum.

Having presented the novel experimental strategy for increasing the yield of the lanthanide anions and determined the EA of Eu, which are the focus of this study, we now turn our attention to other spectroscopic features observed in the photoelectron spectrum (Fig. 2). As shown in Fig. 2, a weaker band, marked as X’, appears at lower binding energy with respect to peak X. The binding energy for this transition is 0.039 eV, which is very close to the energy level of one excited state (^9^P_5_) of Eu^−^ (0.041 eV) calculated by O’Malley and Beck^18^. Thus, this peak most likely originates from this excited state of the anion to the ground state of neutral, establishing the splitting between the ground and excited state of Eu^−^ to be 0.077 eV. To rationalize this identification, one may expect to find transitions coming from this anionic excited state to the excited neutral states in higher binding energy region of the spectrum. Therefore, observation of pair of peaks separated by about 0.077 eV would then be a strong indication of this anionic excited state. After carefully inspecting the spectrum (Fig. 4), six additional peaks, marked as A', A_1_', A_2_', C', C_2_', and B_2_', are found to have such energy interval with respect to their paired peaks originating from the anionic ground state, which are shown in Table 2. This finding provides direct experimental evidence about the existence of this excited state of Eu^−^. In addition, there seem to be several other peaks at higher energy side (up to 0.4 eV) to peak X, which probably come from the transitions between the excited states of Eu^−^ and the excited states of the neutral. The precise assignment of these peaks needs further high-level calculations considering the excited states to make, which is beyond the scope of this study and our ability.

## Discussion

The present study provides the first precise photoelectron imaging spectroscopy of the Eu anion, revealing the character of the electron-atom interaction in Eu^−^. By introducing a new experimental strategy, Eu^−^ with detectable intensity was produced, and the EA was directly measured to be 0.116 ± 0.013 eV, which is in outstanding agreement with the recent high-level theoretical results^18^,^20^. Such a low EA reveals that the additional electron is attached weakly to Eu neutral, resolving the longstanding and significant discrepancy between previous experiment and theory. Moreover, the validation and accuracy of the EA is further verified by comparing the fine structures observed here with the well-established spectroscopic data for neutral. The present experimental results also verify the power of recently advanced theoretical methods in predicting the electronic properties of Eu^−^. For some of other lanthanides, however, significant discrepancy still exists in different theoretical methods with the deviation by factors varying from about 5 to 8^18^,^19^. Thus, to obtain a complete and correct understanding about the lanthanide chemistry, further experiments regarding other lanthanides are urgently desired, which can provide a benchmark to test the accuracy of theory. In fact, we have already acquired the images of several other lanthanides, which will be discussed in other individual works. We believe our experimental findings highlighted here will stimulate further interests and efforts in exploring the fundamental properties of these challenging heavy elements in the periodic table.

## Methods

The Eu^−^ was produced in our laser vaporization source, where a 532 nm second harmonic Nd:YAG laser was used to ablate a 1/4″ Eu “rod” which was made by wrapping an Eu foil around an Al rod. Helium seeded with 5% N_2_O (typically 50 psi) was used as a carrier gas, and the generated Eu^−^ was mass analyzed using a time-of-flight mass spectrometer^44^. Another second harmonic of a Nd:YAG laser (532 nm) was used for photodetaching excess electrons from ^151^Eu^−^. Photoelectrons were accelerated toward position sensitive detectors where the resulting two-dimensional velocity distribution was recorded with a charge-coupled device camera. Then, the three-dimensional distribution was reconstructed from the photoelectron image using the BASEX^45^ and pBASEX^46^ programs, which yielded similar results. The photoelectron spectrum was calibrated against the known Bi^−^ binding energy spectrum^47^.

## Additional Information

**How to cite this article**: Cheng, S.-B. and Castleman, A.W., Jr. Direct experimental observation of weakly-bound character of the attached electron in europium anion. *Sci. Rep*. **5**, 12414; doi: 10.1038/srep12414 (2015).

## Figures and Tables

**Table 1:** Atomic energy levels (in eV) in Eu^0/−^.

Band	Binding energy (eV)	Atomic energy level (eV) (this work)	Atomic energy level (eV) (Ref. 43)	Term of final state
X'	0.039	−0.077	—	—
X	0.116	0	0	^8^ *S*_7/2_
A	1.725	1.609	1.602	^10^ *D*_5/2_
A_1_	1.746	1.630	1.618	^10^ *D*_7/2_
A_2_	1.773	1.657	1.639	^10^ *D*_9/2_
A_3_	1.797	1.681	1.668	^10^ *D*_11/2_
A_4_	1.830	1.718	1.708	^10^ *D*_13/2_
B	1.864	1.748	1.744	^10^ *P*_7/2_
B_1_	1.910	1.794	1.806	^10^ *P*_9/2_
C	1.995	1.879	1.877	^8^ *D*_3/2_
C_1_	2.009	1.893	1.891	^8^ *D*_5/2_
C_2_	2.024	1.908	1.912	^8^ *D*_7/2_
B_2_	2.044	1.928	1.932	^10^ *P*_11/2_
C_3_	2.063	1.947	1.944	^8^ *D*_9/2_
D	2.088	1.972	1.970	^8^ *P*_5/2_
E	2.261	2.145	2.150	^6^ *P*_7/2_

Experimental binding energies (BEs) have an uncertainty of ±0.013 eV. The present atomic energy levels are obtained by calculating the energy differences between peak X and other peaks observed in the photoelectron spectrum. The referenced spectroscopic data are obtained from Ref. 43.

**Table 2:** Observed transitions originating from one excited state of Eu^−^, in eV.

Band	Binding energy (eV)	Paired peak	Binding energy (eV)	ΔE (eV)
A'	1.650	A	1.725	0.075
A_1_'	1.672	A_1_	1.746	0.074
A_2_'	1.699	A_2_	1.773	0.074
C'	1.920	C	1.995	0.075
C_2_'	1.950	C_2_	2.024	0.074
B_2_'	1.967	B_2_	2.044	0.077

Experimental binding energies (BEs) have an uncertainty of ± 0.013 eV. ΔE represent the energy differences between peaks A' and A, A_1_' and A_1_, A_2_' and A_2_ and so on.
